# Physical Activity and Depressive Symptoms in Guangdong University Students: A Cross-Sectional Moderated Serial Indirect-Association Model Involving Self-Esteem, Positive Psychological Capital, and Perceived Social Support

**DOI:** 10.3390/healthcare14203484

**Published:** 2026-10-16

**Authors:** Zhihui Liu, Zhijie Lai, Taufik Rihatno, Widiastuti, Johansyah Lubis

**Affiliations:** 1 Faculty of Sport Science, Universitas Negeri Jakarta, East Jakarta 13220, Indonesia; 2 Physical Education Department, Hubei University of Technology Engineering and Technology College, Wuhan 430068, China; 3 School of Physical Education, Guangzhou College of Commerce, Guangzhou 511363, China; 4 College of Exercise and Health, Guangzhou Sport University, Guangzhou 510500, China

**Keywords:** physical activity, depressive symptoms, self-esteem, positive psychological capital, perceived social support, university students

## Abstract

**Background:** This cross-sectional study examined whether physical activity was indirectly associated with depressive symptoms through self-esteem and positive psychological capital and whether these associations varied by perceived social support. Methods: Data from 473 students at three Guangdong universities were used to assess physical activity, depressive symptoms, self-esteem, positive psychological capital, and perceived social support. Serial and moderated serial indirect associations were estimated using PROCESS v4.1 (Model 6; a custom moderated specification equivalent to PROCESS Model 89 for the joint outcome equation; 5000 bootstrap resamples), adjusting for gender, grade, and hometown, with three interactions entered simultaneously in the same outcome equation. **Results:** Physical activity was negatively associated with depressive symptoms (total association: B = −0.175, 95% CI [−0.215, −0.138]; direct association: B = −0.079, 95% CI [−0.120, −0.039]), and the total indirect association was B = −0.097 (95% CI [−0.126, −0.070]), with specific indirect associations through self-esteem (B = −0.031), positive psychological capital (B = −0.048), and the serial pathway (B = −0.017); the positive psychological capital pathway was significantly larger than the serial pathway (difference = −0.031, 95% CI [−0.048, −0.016]). Perceived social support moderated the associations of physical activity (B = 0.0043, *p* = 0.009), self-esteem (B = 0.0256, *p* < 0.001), and positive psychological capital (B = 0.0062, *p* < 0.001) with depressive symptoms in the simultaneous custom moderated outcome equation (R^2^ = 0.449); all three indices of moderated mediation were significant, and conditional indirect associations were stronger at lower support, with the self-esteem pathway no longer significant at high support. **Conclusions:** Physical activity was cross-sectionally associated with fewer depressive symptoms via independent and serial indirect associations involving self-esteem and positive psychological capital, with perceived social support showing a compensatory moderating pattern; temporal or causal ordering was not established.

## 1. Introduction

Depressive symptoms are a major mental health concern among university students worldwide. The transition to adulthood often coincides with academic pressure, changes in social relationships, employment uncertainty, and increasing personal responsibility, all of which may heighten vulnerability to psychological distress. In China, a recent meta-analysis estimated that approximately one-third of university students experience scale-defined depressive symptoms, although the prevalence varies across populations and assessment instruments [1]. Such symptoms are associated with impaired academic functioning, reduced quality of life, and an increased risk of persistent mental health difficulties. Identifying accessible and scalable factors associated with lower depressive symptom severity is therefore important for both university health promotion and public health practice.

Physical activity (PA) is increasingly recognized as a potentially modifiable correlate of mental health. Prospective and intervention-based evidence indicates that greater PA and structured exercise are associated with a lower risk of depression and reduced depressive symptoms [2,3,4]. PA is particularly relevant in university settings because it can be incorporated into physical education, campus recreation, and students’ daily routines. However, evidence of an inverse association between PA and depressive symptoms does not fully explain the psychological processes involved. Understanding these processes may help universities move beyond general exercise promotion toward more targeted strategies that combine behavioral participation with psychological resource development.

Self-esteem may represent one pathway linking PA with depressive symptoms. It reflects an individual’s overall evaluation of personal worth and competence. Participation in PA may provide opportunities for mastery, goal attainment, improved physical self-perception, and social recognition, which are associated with more positive self-evaluations [5]. Longitudinal evidence also suggests that low self-esteem is a vulnerability factor for subsequent depressive symptoms [6]. Accordingly, self-esteem may statistically account for part of the association between PA and depressive symptoms.

Positive psychological capital (PsyCap) may constitute a complementary pathway. PsyCap is a higher-order psychological resource comprising self-efficacy, hope, optimism, and resilience. Experiences of mastery, persistence, and successful coping during PA may be associated with the development of these resources. PsyCap is also inversely associated with depressive symptoms, although much of the available evidence is cross-sectional [7]. Previous research has suggested that among university students, self-esteem and PsyCap may operate as both independent and sequential variables between PA and depressive symptoms [8]. Theoretically, self-esteem reflects a relatively broad and global evaluation of personal worth, whereas PsyCap represents more future-oriented psychological resources, including self-efficacy, hope, optimism, and resilience. Because global self-evaluations may provide a foundation for these future-oriented resources, positive self-evaluations may support stronger efficacy beliefs, goal-directed motivation, optimism, and resilience, providing a rationale for specifying a serial indirect pathway from self-esteem to PsyCap. However, because self-esteem and PsyCap were assessed concurrently, the proposed ordering from self-esteem to PsyCap is theoretically imposed rather than empirically established. The present cross-sectional data cannot determine whether self-esteem precedes PsyCap, PsyCap precedes self-esteem, or whether the two constructs are reciprocally related.

These psychological resources operate within a broader social context. Perceived social support refers to the belief that emotional, informational, and practical assistance is available from family, friends, and significant others. According to the stress–buffering perspective, social support may protect mental health by reducing the psychological consequences of stress [9]. Previous studies have linked social support with PA participation and have examined it as a mediator between PA and psychological outcomes [10]. Nevertheless, social support may also serve as a boundary condition. Greater perceived support could strengthen the associations of PA and psychological resources with depressive symptoms, or it could compensate for limited behavioral and intrapersonal resources, thereby weakening their relative associations with depression.

**Figure 1:**
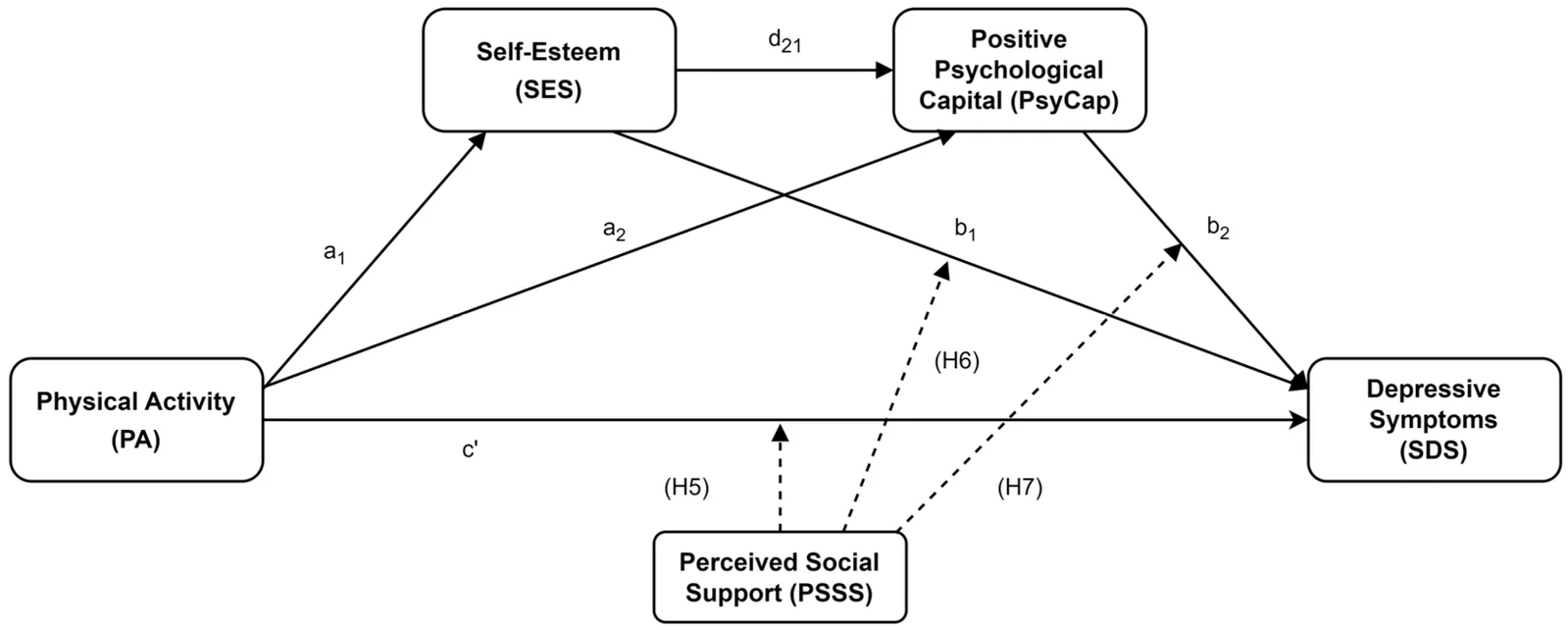
Hypothesized moderated serial indirect-association model. Solid arrows represent the hypothesized associations among physical activity, self-esteem, positive psychological capital, and depressive symptoms. Dashed arrows indicate the hypothesized moderating effects of perceived social support on the associations of physical activity, self-esteem, and positive psychological capital with depressive symptoms. PA, physical activity; SES, self-esteem; PsyCap, positive psychological capital; PSSS, perceived social support; SDS, Self-Rating Depression Scale. The letters (a_1_, a_2_, b_1_, b_2_, c′, d_21_) represent the path coefficients.

To address these gaps, the present study tested a cross-sectional moderated serial indirect-association model among university students recruited from three universities in Guangdong Province, China (Figure 1). We examined whether physical activity was negatively associated with depressive symptoms (H1) and whether physical activity was indirectly associated with depressive symptoms through self-esteem (H2), positive psychological capital (PsyCap; H3), and the serial pathway from self-esteem to PsyCap (H4). We further tested whether perceived social support moderated the associations of physical activity (H5), self-esteem (H6), and PsyCap (H7) with depressive symptoms, as well as the serial indirect association through self-esteem and PsyCap (H8). Given the cross-sectional design, the proposed serial ordering represents a theory-constrained statistical specification rather than an empirically established temporal or causal sequence.

## 2. Materials and Methods

### 2.1. Study Design, Participants, and Procedure

A cross-sectional survey was conducted among undergraduate students from three universities in Guangdong Province, China, between March and May 2025. First- to third-year students were invited to participate through class announcements and online platforms. Age information, university-specific identifiers, class-level identifiers, and recruitment-mode counts were not retained in the final analytic dataset. Therefore, age-related descriptive statistics, the number of analyzed participants contributed by each university or class, and the distribution of participants across recruitment channels could not be reconstructed. Potential clustering of observations within universities or classes could not be formally evaluated or adjusted for. Participation was voluntary and uncompensated. The purpose and procedures of the study were explained to all participants verbally and in writing, and written informed consent was obtained before questionnaire completion. The questionnaires were completed anonymously, and no personally identifying information was collected.

An a priori sample-size calculation was performed using G*Power version 3.1.9.7. The most complex outcome regression included 10 predictors: physical activity, self-esteem, positive psychological capital, perceived social support, three interaction terms, and three demographic covariates. For a fixed-effects multiple regression model with a medium effect size of f^2^ = 0.15, α = 0.05, a power of 0.95, and 10 predictors, the minimum required sample was 172 participants. The final sample (*n* = 473) exceeded the minimum sample size estimated for the overall multiple regression model; this calculation was based on the overall regression effect and was not specifically designed to establish statistical power for the smallest interaction or conditional indirect effect [11].

A total of 670 questionnaires were returned. During data screening, 197 questionnaires were identified as incomplete because one or more required questionnaire items had not been answered. These incomplete questionnaires were excluded in their entirety, and no item-level missing values were imputed. Accordingly, only questionnaires with complete data on all variables included in the analyses were retained. The final analytic sample consisted of 473 participants, corresponding to a usable-return proportion of 70.60% (473/670). The final sample comprised 105 men and 368 women. A total of 398 participants were first–year students, whereas 75 were second– or third–year students. With respect to hometown location, 179 students were from urban areas, and 294 were from rural areas. The study was conducted in accordance with the Declaration of Helsinki and was approved by the Human Ethics Committee of the Research Division at Guangzhou Sport University (Approval No. 2025LCLL-182).

During the data audit conducted for the present revision, the PARS-3 total score was re-examined for extreme values. To reduce the potential influence of extreme observations, values below the 1st percentile and above the 99th percentile were Winsorized to the corresponding percentile cut points. Eight cases were Winsorized (four below the 1st percentile and four above the 99th percentile; cut points = 2.0 and 96.0). No participants were removed, so the analytic sample remained *n* = 473. This Winsorization procedure was introduced during the revision-stage data audit and was not preregistered. All descriptive statistics and regression analyses involving physical activity reported in the revised manuscript are based on the Winsorized PARS-3 score. Results using the untransformed PARS-3 score are provided as a sensitivity analysis in Table S3.

### 2.2. Measurements

The continuous total scores of all the instruments were used in the primary analyses. Higher scores represented higher levels of the corresponding constructs.

#### 2.2.1. Physical Activity

Physical activity was assessed using the Physical Activity Rating Scale-3 [12]. The scale comprises three items assessing exercise intensity, duration, and frequency. Intensity and frequency are each scored from 1 to 5, whereas duration is coded from 0 to 4. The total physical activity score was calculated as follows: PARS-3 score = intensity × duration × frequency. Total scores range from 0 to 100, with higher scores indicating greater physical activity. Although the PARS-3 score may be classified as low, moderate, or high, the continuous total score was used in the present analyses to avoid loss of information. Because the PARS-3 is a multiplicatively scored composite index, it was treated as an observed composite variable rather than as a reflective latent construct. Accordingly, internal consistency, composite reliability, and average variance extracted were not computed for the PARS-3, and its three components were not included as reflective indicators in the confirmatory factor analysis.

#### 2.2.2. Depressive Symptoms

Depressive symptoms were assessed using the 20-item Zung Self-Rating Depression Scale (SDS) [13]. The Chinese version of the SDS, which has demonstrated acceptable reliability and validity among Chinese university students, was used [14]. Items are rated on a 4-point response scale according to the frequency of each symptom. Positively and negatively worded items were scored in accordance with the original scoring instructions. The scores for the 20 items were summed to produce a raw score ranging from 20 to 80, which was multiplied by 1.25 to obtain an index score ranging from 25 to 100. Higher scores indicated more severe depressive symptoms.

The SDS score was analyzed as a continuous measure and was not treated as a clinical diagnosis of depressive disorder. Cronbach’s α was 0.923 in the current sample.

#### 2.2.3. Self-Esteem

Global self-esteem was measured using the 10-item Rosenberg Self-Esteem Scale (SES) [15]. The Chinese version has demonstrated satisfactory factorial and criterion validity among Chinese undergraduate students [16]. Items are rated on a 4-point scale ranging from 1 (strongly disagree) to 4 (strongly agree). Negatively worded items were reverse scored before all item scores were summed. Total scores range from 10 to 40, with higher scores indicating greater global self-esteem. Cronbach’s α was 0.895 in the current sample.

#### 2.2.4. Positive Psychological Capital

Positive psychological capital was assessed using the 26-item Positive Psychological Capital Questionnaire (PPQ) [17]. The questionnaire assesses four dimensions: self-efficacy, hope, resilience, and optimism. Items are rated on a 7-point scale ranging from 1 (completely inconsistent) to 7 (completely consistent). Items were scored according to the original instructions and summed to produce a total score, with higher scores indicating greater positive psychological capital. The Cronbach’s α was 0.974 in the current sample. Although the PPQ is a multidimensional instrument, the present study focused on its total score as an indicator of global positive psychological capital. The one-first-order-factor specification was therefore intended to evaluate the total-score composite, not to test the full multidimensional structure. Given the mixed incremental fit (CFI/TLI/IFI below 0.90), this unidimensional representation should be regarded as approximate, and the total score should be interpreted cautiously. The same approach was applied to the PSSS, which is also multidimensional but was modeled as a single first-order factor representing the global perceived social support composite.

#### 2.2.5. Perceived Social Support

Perceived social support was measured using the 12-item Multidimensional Scale of Perceived Social Support (MSPSS) [18]. The scale is also referred to as the Perceived Social Support Scale (PSSS) in some Chinese adaptations. The Chinese version has demonstrated good reliability and validity among Chinese college students [19]. The scale assesses perceived support from family, friends, and significant others, with four items representing each source. Items are rated on a 7-point scale ranging from 1 (very strongly disagree) to 7 (very strongly agree). The item scores were summed to produce a total score ranging from 12 to 84, with higher scores indicating greater perceived social support. The Cronbach’s α was 0.938 in the current sample.

### 2.3. Statistical Analysis

Statistical analyses were performed using IBM SPSS Statistics 26.0. Confirmatory factor analysis was conducted using the Python package semopy 2.3.11. For the CFA, all 68 psychological items were specified as first-order indicators of their respective factors: SES (10 items), PPQ (26 items), SDS (20 items), and PSSS (12 items). No second-order or parcel structures were specified. Total scores were used only in the regression analyses. Means, standard deviations, Pearson correlations, Cronbach’s α, composite reliability, average variance extracted, and variance inflation factors were calculated. Because the PARS-3 was treated as an observed composite index, confirmatory factor analysis and latent-variable validity analyses focused on the psychological scales (SES, PPQ, SDS, and PSSS). Confirmatory factor analysis was used to compare the hypothesized four-factor model with one-factor, two-factor, and three-factor alternative models. Model fit was evaluated using the χ^2^/df, CFI, TLI, IFI, RMSEA, and SRMR. Potential common method variance was assessed using Harman’s single-factor test and a confirmatory factor model including a common method factor. The common method factor loaded on all 68 items.

Before the main analyses, the continuous predictors used in the interaction terms (physical activity, self-esteem, positive psychological capital, and perceived social support) were mean-centered before the product terms were created. Gender was coded as 0 = female and 1 = male; grade was coded as 0 = first-year and 1 = second- or third-year; hometown location was coded as 0 = rural and 1 = urban. These coded variables were included as covariates. Gender, grade, and hometown location were included as covariates because they may be associated with physical activity and depressive symptoms and could therefore act as potential confounders.

Serial indirect associations were estimated using PROCESS version 4.1, Model 6, with physical activity as the independent variable, self-esteem and positive psychological capital as sequential intermediate variables, and depressive symptoms as the outcome. The serial ordering from self-esteem to positive psychological capital was theory-specified based on theoretical considerations and does not imply an empirically established temporal or causal sequence. The study was not preregistered. H1–H8 were theory-informed hypotheses rather than preregistered confirmatory hypotheses. The corresponding regression equations were specified as follows:

SES = a_0_ + a_1_(PA) + Σa_k_(Covariates) + ε_1_

PsyCap = a_0_′ + a_2_(PA) + d_21_(SES) + Σa_k_(Covariates) + ε_2_

For the moderated serial indirect-association analysis, perceived social support was specified as the moderator in the depressive-symptom outcome equation. Because default PROCESS templates do not simultaneously estimate all three focal interactions in the same outcome equation, the model was implemented as a custom moderated specification in PROCESS v4.1. This custom specification had the same path structure as PROCESS Model 89 but was adapted to estimate the PA × PSSS, SES × PSSS, and PsyCap × PSSS interactions simultaneously in the same depressive-symptom outcome equation. We therefore refer to this analysis throughout as the custom moderated specification equivalent to PROCESS Model 89. The focal joint outcome equation was specified as:

SDS = b_0_ + c′(PA) + b_2_(SES) + b_3_(PsyCap) + b_4_(PSSS) + i_1_(PA × PSSS) + i_2_(SES × PSSS) + i_3_(PsyCap × PSSS) + Σb_k_(Covariates) + ε_3_

Thus, all three interaction terms were estimated simultaneously within the same depressive-symptom outcome equation. Continuous predictors involved in the interaction terms were mean-centered before creation of the product terms. Gender, grade, and hometown location were included as covariates. Indirect and conditional indirect associations were estimated using 5000 bootstrap resamples with percentile-based 95% bootstrap confidence intervals. Simple slopes were estimated as unstandardized conditional coefficients from the custom moderated outcome equation, and their 95% confidence intervals were based on model standard errors. These associations were considered statistically significant when the corresponding confidence interval excluded zero. *n* = 473 for each equation. No automatic correction for multiple testing was applied, and the confidence intervals were not adjusted for multiple testing. Exact *p* values are reported where available; *p* < 0.001 is reported when the exact value is below this threshold. Given the number of tested paths and the cross-sectional design, these results should be interpreted as theory-informed and exploratory rather than definitive confirmatory evidence. Regression diagnostics for the focal outcome model included inspection of standardized residuals and residual-versus-predicted plots. The maximum studentized residual was 3.709, with one case exceeding |z| > 3; excluding this case did not change the direction or significance of any focal effect. The leverage threshold used was 2(k + 1)/*n* = 0.0465; the maximum leverage was 0.153, and 42 cases exceeded this screening threshold, but all had Cook’s D < 0.06. The maximum Cook’s D was 0.056, below the conventional threshold of 1. Residual-versus-predicted plots showed no clear nonlinearity or heteroscedasticity. No influential observations materially altered the results.

## 3. Results

### 3.1. Reliability and Measurement-Model Results

Because the PARS-3 was treated as an observed composite index, reliability and latent-variable validity statistics are reported only for the psychological scales. The Cronbach’s α coefficients ranged from 0.895 to 0.974, and the composite reliability values ranged from 0.897 to 0.974, indicating acceptable internal consistency. The standardized factor loadings ranged from 0.584 to 0.818. The average variance extracted was 0.466 for self-esteem, 0.592 for positive psychological capital, 0.390 for depressive symptoms, and 0.561 for perceived social support. Although the AVE for self-esteem and depressive symptoms was below 0.50, their composite reliability values were 0.897 and 0.927, respectively, supporting acceptable internal consistency. The square roots of AVE were 0.682 for self-esteem, 0.769 for positive psychological capital, 0.625 for depressive symptoms, and 0.749 for perceived social support. The square root of the average variance extracted for each construct exceeded its absolute latent correlation with the other constructs, supporting discriminant validity. Table 1 no longer reports factor loadings, Cronbach’s α, composite reliability, or AVE for PARS-3; only its variance inflation factor is reported as an observed predictor.

### 3.2. Measurement Model, Common Method Variance, and Multicollinearity

Harman’s single-factor analysis revealed that the first unrotated factor accounted for 30.82% of the total variance, indicating that no single factor dominated the measurement variance.

As shown in Table 2, the four-factor model fit the data better than the one-factor, two-factor, and three-factor alternative models, although its incremental fit indices remained below 0.90: χ^2^/df = 2.524, IFI = 0.845, TLI = 0.839, CFI = 0.845, RMSEA = 0.057, and SRMR = 0.043. The one-factor model showed substantially poorer fit (χ^2^/df = 5.577, IFI = 0.534, TLI = 0.518, CFI = 0.532, RMSEA = 0.098, and SRMR = 0.125). The two-factor models also fit worse than the four-factor model (Model a: χ^2^/df = 4.518, CFI = 0.641, RMSEA = 0.086; Model b: χ^2^/df = 4.491, CFI = 0.643, RMSEA = 0.086), as did the three-factor model (χ^2^/df = 3.159, CFI = 0.780, RMSEA = 0.068).

For the common method factor, the method factor loaded on all 68 items. In the non-orthogonal specification, the method-factor covariances with the four trait factors (SES, PPQ, SDS, PSSS) were freely estimated, yielding df = 2132, χ^2^ = 5384.97, CFI = 0.850, TLI = 0.839, RMSEA = 0.057, and SRMR = 0.042. In the orthogonal sensitivity model, these four covariances were fixed to zero and the method-factor variance was fixed to 1, yielding df = 2137, χ^2^ = 5583.35, CFI = 0.841, TLI = 0.830, and RMSEA = 0.058. The difference in df between the four-factor and non-orthogonal method-factor models was 72, which is explained by the 68 method-factor loadings plus the 4 trait–method covariances. Both method-factor models produced estimation warnings (negative variance/non-positive-definite information matrix) in semopy and lavaan; they are therefore reported as a supplementary check on common method variance rather than as primary evidence.

Variance inflation factors ranged from 1.120 to 1.537, indicating no evidence of problematic multicollinearity. The four-factor model was retained as the final measurement model for subsequent analyses. However, its incremental fit indices (CFI, TLI, IFI) were below 0.90, whereas RMSEA and SRMR were acceptable. Thus, the four-factor model received relative support over the competing one-, two-, and three-factor models, but the absolute model fit remained mixed and the incremental fit indices did not reach conventional thresholds. These measurement-model findings should therefore be interpreted cautiously.

### 3.3. Descriptive Statistics and Correlations

Descriptive statistics and Pearson correlations are presented in Table 3. Physical activity was positively correlated with self-esteem (r = 0.323), positive psychological capital (r = 0.438), and perceived social support (r = 0.145); all *p* < 0.01. Self-esteem was positively correlated with positive psychological capital (r = 0.465), and perceived social support was positively correlated with self-esteem (r = 0.242) and positive psychological capital (r = 0.304); all *p* < 0.01.

Physical activity (r = −0.369), self-esteem (r = −0.398), positive psychological capital (r = −0.475), and perceived social support (r = −0.255) were negatively correlated with depressive symptoms (all *p* < 0.01).

### 3.4. Serial Mediation Analysis

The results of the serial indirect-association analysis are presented in Table 4. After adjustment for gender, grade, and hometown location, physical activity was negatively associated with depressive symptoms (total association: B = −0.175, 95% CI [−0.215, −0.138]). When self-esteem and positive psychological capital were included as intermediate variables, the direct association remained significant (B = −0.079, 95% CI [−0.120, −0.039]), indicating a pattern consistent with partial statistical indirect association rather than causal mediation. The total indirect association was also significant (B = −0.097, bootstrap SE = 0.014, 95% CI [−0.126, −0.070]).

All three specific indirect associations were statistically significant. Physical activity was indirectly associated with depressive symptoms through self-esteem (B = −0.031, bootstrap SE = 0.009, 95% CI [−0.049, −0.016]), through positive psychological capital (B = −0.048, bootstrap SE = 0.009, 95% CI [−0.068, −0.031]), and sequentially through self-esteem and positive psychological capital (B = −0.017, bootstrap SE = 0.004, 95% CI [−0.026, −0.010]). The indirect associations through self-esteem and positive psychological capital did not differ significantly (B = 0.017, 95% CI [−0.009, 0.043]). Compared with the serial indirect pathway, the positive psychological capital pathway was significantly greater in absolute magnitude (B = −0.031, 95% CI [−0.048, −0.016]), whereas the self-esteem pathway did not differ significantly from the serial pathway (B = −0.014, 95% CI [−0.032, 0.003]). These results were consistent with H1–H4 in this cross-sectional sample.

### 3.5. Primary Moderated Serial Indirect-Association Analysis

The primary moderated serial indirect-association analysis included physical activity, self-esteem, positive psychological capital, perceived social support, and the three interaction terms (PA × PSSS, SES × PSSS, and PsyCap × PSSS) simultaneously in the depressive-symptom outcome equation, with gender, grade, and hometown included as covariates. The coefficients are reported in Table 5. All continuous predictors were mean-centered before the product terms were created.

As shown in Table 5, physical activity was positively associated with self-esteem (B = 0.083, *p* < 0.001) and positive psychological capital (B = 0.390, *p* < 0.001), and self-esteem was positively associated with positive psychological capital (B = 1.647, *p* < 0.001). In the simultaneous moderated SDS outcome equation, all three interaction terms were statistically significant: PA × PSSS (B = 0.0043, *p* = 0.009), SES × PSSS (B = 0.0256, *p* < 0.001), and PPQ × PSSS (B = 0.0062, *p* < 0.001). The simultaneous model accounted for 44.9% of the variance in depressive symptoms (R^2^ = 0.449, adjusted R^2^ = 0.437, F(10, 462) = 37.589, *p* < 0.001, *n* = 473).

Because the main associations of physical activity, self-esteem, and positive psychological capital with depressive symptoms were negative, the positive interaction coefficients indicate an attenuating pattern: the negative associations became weaker as perceived social support increased. These findings were consistent with H5–H7.

### 3.6. Conditional Indirect Associations and Simple Slopes

Formal indices of moderated mediation were examined first. As shown in Table 6, all three bootstrap confidence intervals excluded zero, indicating that the corresponding indirect associations varied as a function of perceived social support.

Conditional indirect associations at low, mean, and high levels of perceived social support are summarized in Table 7. The absolute magnitudes of all three negative indirect associations decreased as PSSS increased. The indirect association through self-esteem was no longer statistically significant at high PSSS, whereas the PsyCap-only and serial indirect associations remained statistically significant.

Simple-slope analyses further clarified the attenuating pattern (Table 8). The associations of physical activity and self-esteem with depressive symptoms were statistically significant at low and mean PSSS but not at high PSSS. The association of positive psychological capital with depressive symptoms remained statistically significant at all three PSSS levels, although its absolute magnitude decreased as PSSS increased.

**Figure 2:**
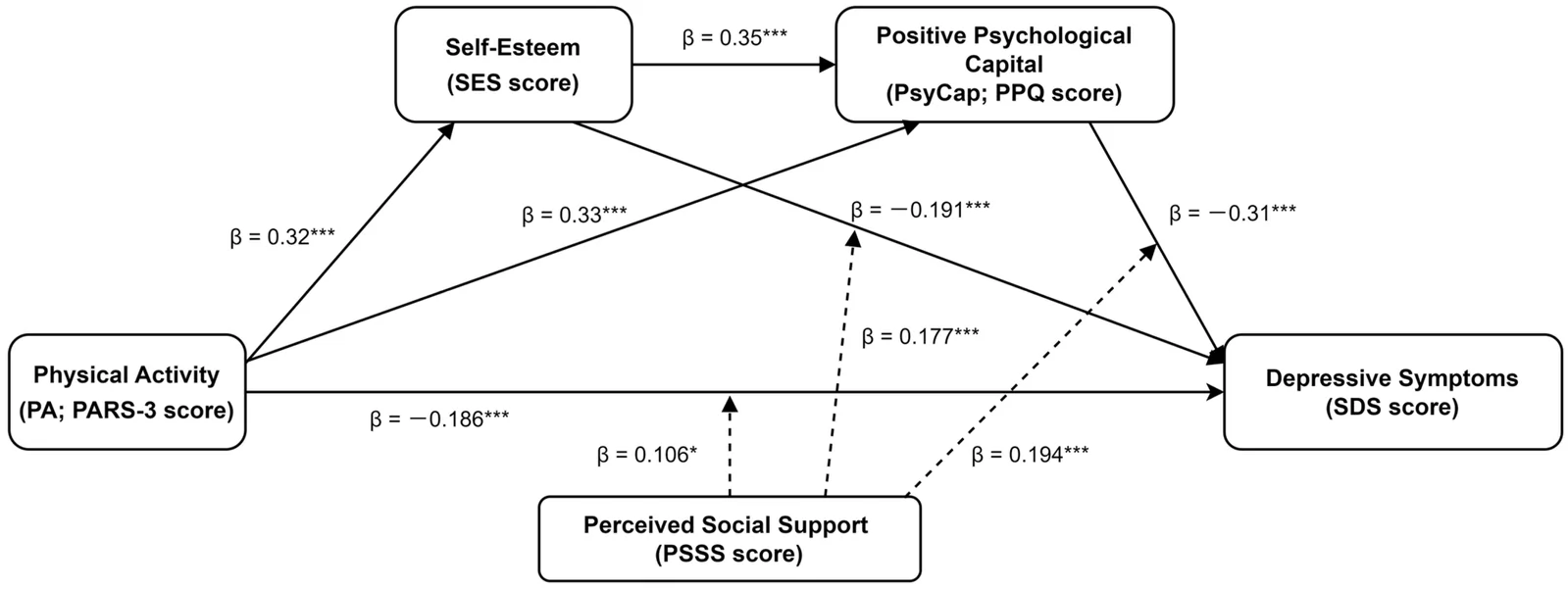
Standardized path coefficients for the moderated serial indirect-association model. Coefficients for the PA → SES, PA → PsyCap, and SES → PsyCap paths were obtained from the two intermediate regression equations. Coefficients for the PA → SDS, SES → SDS, PsyCap → SDS, and interaction paths were obtained from the simultaneous depressive-symptom outcome equation. PA, physical activity; SES, self-esteem; PsyCap, positive psychological capital; PSSS, perceived social support; SDS, depressive symptoms. The PSSS → SDS main effect (β = −0.098, *p* = 0.007) is not shown for clarity. * *p* < 0.05, *** *p* < 0.001.

Overall, the conditional indirect associations decreased in absolute magnitude as perceived social support increased. Together with the significant indices of moderated mediation, this pattern was consistent with the moderated indirect association proposed in H8. These results describe variation in cross-sectional statistical associations and should not be interpreted as evidence of differential causal effects across levels of perceived social support (Figure 2).

## 4. Discussion

Physical activity was associated with fewer depressive symptoms directly and through independent and serial indirect associations involving self-esteem and positive psychological capital. The positive psychological capital pathway was larger in absolute magnitude than the serial pathway, whereas the self-esteem pathway did not differ significantly from the serial pathway.

### 4.1. Physical Activity and Depressive Symptoms

The negative association between physical activity and depressive symptoms is consistent with previous evidence that physically active individuals generally report better mental health. Meta-analytic findings indicate that exercise is associated with a reduction in depressive symptoms across different populations and exercise modalities [2,3,4,20]. The present study extends this evidence to Chinese university students from the three sampled universities in Guangdong, who often experience simultaneous academic, interpersonal, and employment-related demands.

Several psychosocial processes may help explain this association. Physical activity provides opportunities for behavioral activation, mastery, goal attainment, social interaction, and temporary disengagement from stress-related thoughts. Such experiences may enhance perceived competence and support more adaptive emotion regulation. Participation in structured activity can also provide routine and achievement experiences, which may be particularly relevant during periods of academic uncertainty or psychological distress.

However, self-esteem and PsyCap did not fully account for the association between physical activity and depressive symptoms. The remaining direct association suggests that other pathways may also be involved, including social connection, sleep, emotion regulation, and physiological responses to activity. Because these factors were not measured, their roles cannot be established from the present data.

### 4.2. Independent Roles of Self-Esteem and Positive Psychological Capital

Self-esteem statistically accounted for part of the association between physical activity and depressive symptoms. Physical activity can provide competence-related information through skill acquisition, improved performance, physical functioning, and social recognition. These experiences may contribute to a more favorable evaluation of personal worth, particularly when participation emphasizes individual progress rather than comparisons with others.

These findings are consistent with those of [21], who identified self-esteem and self-efficacy as psychological mechanisms associated with the relationship between physical activity and depressive symptoms. They also align with evidence that improvements in physical self-concept through exercise participation correspond with greater global self-esteem and mental well–being [22]. Research involving young people has similarly suggested that exercise participation can support self-esteem, although the magnitude and certainty of these effects vary across study designs and populations [23,24].

Positive psychological capital also independently accounted for part of the association between physical activity and depressive symptoms. PsyCap comprises self-efficacy, hope, optimism, and resilience, all of which are relevant to coping with academic and interpersonal demands. Physical activity repeatedly exposes individuals to goal setting, effort, temporary failure, persistence, and improvement. These experiences are conceptually consistent with stronger efficacy beliefs, goal-directed motivation, positive expectations, and adaptive responses to setbacks.

Previous research has associated higher PsyCap with better psychological adjustment among university students and has suggested that interventions targeting PsyCap may reduce depressive symptoms [7,25,26]. The current findings therefore support PsyCap as a relevant psychological resource in the association between physical activity and depressive symptoms. Nevertheless, the cross-sectional design does not demonstrate that physical activity increased PsyCap or that greater PsyCap subsequently reduced depressive symptoms.

Compared with the serial indirect pathway, the independent indirect association through positive psychological capital was significantly larger in absolute magnitude, whereas the self-esteem pathway did not differ significantly from the serial pathway.

### 4.3. Serial Association Through Self-Esteem and Positive Psychological Capital

Self-esteem and PsyCap showed a significant serial indirect association. Conceptually, self-esteem reflects an individual’s overall evaluation of personal worth, whereas PsyCap reflects confidence and future-oriented resources for managing challenges. A more positive self-evaluation may provide a foundation for stronger efficacy beliefs, hope, optimism, and resilience. Self-esteem and PsyCap should therefore be viewed as related but distinct psychological resources.

The significant serial indirect association should not be interpreted as evidence that physical activity first changes self-esteem, which subsequently changes PsyCap and then depressive symptoms. Because the variables were measured concurrently, the proposed sequence is consistent with the observed statistical associations but does not establish temporal ordering. Reverse or reciprocal relationships remain possible.

These findings are consistent with Wei et al. [8], who reported independent and serial indirect associations of self-esteem and PsyCap in the association between physical activity and depressive symptoms among university students. The present study further extends this model by examining perceived social support as a boundary condition.

The serial indirect association was numerically smaller than both independent indirect associations. However, only the difference from the positive psychological capital pathway was statistically significant (B = −0.031, 95% CI [−0.048, −0.016]); the difference from the self-esteem pathway was not significant (B = −0.014, 95% CI [−0.032, 0.003]). This suggests that the psychological correlates of physical activity are not organized exclusively as a single linear sequence. Physical activity may be associated with self-esteem and PsyCap through partly distinct experiences, whereas self-esteem may additionally be associated with PsyCap. The results therefore support a model involving both parallel and sequential psychological pathways rather than one dominant mechanism.

Related conditional process studies have also shown that physical activity is associated with university students’ psychological outcomes through the use of multiple personal and social resources [10,27,28]. The present findings add to this literature by distinguishing the independent and serial roles of self-esteem and PsyCap while incorporating perceived social support as a moderator.

### 4.4. Compensatory Role of Perceived Social Support

Perceived social support attenuated the negative associations of physical activity, self-esteem, and positive psychological capital with depressive symptoms, although the moderation effect for physical activity was relatively small (B = 0.0043, *p* = 0.009). The primary moderated analysis estimated all three interaction terms simultaneously in the same outcome equation. Because the main associations of these three predictors with depressive symptoms were negative, the positive interaction coefficients indicated that their negative associations became weaker as perceived social support increased. All three conditional indirect associations were smaller in absolute magnitude at higher levels of social support. At high perceived social support, the indirect association through self-esteem was no longer significant, whereas the indirect associations through positive psychological capital and the serial pathway remained significant but attenuated.

This does not imply that social support is harmful. Perceived social support was negatively correlated with depressive symptoms, suggesting that students who felt more supported generally reported fewer symptoms. Instead, the interaction pattern is consistent with resource compensation. When students already perceive reliable emotional, informational, and practical support, the relative association of physical activity or intrapersonal resources with depressive symptoms may be weaker. In contrast, physical activity, self-esteem, and PsyCap may be more salient among students whose interpersonal resources are limited.

This interpretation is compatible with the view that social relationships contribute to health through overlapping behavioral and psychological pathways [29]. Social support may provide emotional reassurance, practical assistance, belonging, and alternative coping resources. When these resources are available, students may depend less strongly on physical activity or personal psychological resources in relation to depressive symptoms.

These findings differ from the original interpretation that social support strengthened the beneficial associations of physical activity, self-esteem, and PsyCap. The conditional estimates showed the opposite pattern: negative indirect associations were stronger under lower support. A similar context-dependent pattern was reported by Zuo et al. [28], who found that the association between physical activity and negative emotions varied according to supportive resources.

Other studies have treated social support as a facilitator of physical activity or as an intervening variable that links physical activity with psychological outcomes [10,30,31]. These perspectives are not necessarily contradictory. Social support can be positively associated with activity participation, is independently associated with mental health, and simultaneously alters the relative importance of physical activity and intrapersonal resources. Its role depends on its position within the theoretical model and the outcome being examined.

The moderated indirect associations further indicate that the relevance of self-esteem and PsyCap varies across social contexts. Under lower perceived support, the indirect associations through both resources were stronger. Under higher support, the self-esteem pathway was no longer significant, and the physical activity and self-esteem simple slopes also became non-significant. The positive psychological capital pathway and the serial pathway remained significant but were substantially attenuated. These findings support a contextualized resource model in which intrapersonal strengths are particularly salient when interpersonal resources are limited. Longitudinal evidence is needed before this pattern can be interpreted as a developmental or causal process.

### 4.5. Implications, Strengths, and Limitations

Theoretically, the findings integrate behavioral engagement, self-evaluation, future-oriented psychological resources, and interpersonal support within one conditional process model. The findings also indicate that psychological resources do not necessarily accumulate in a uniformly additive manner: self-esteem and PsyCap showed independent and serial indirect associations, whereas perceived social support reduced the magnitude of these associations. This pattern supports a context-sensitive rather than purely cumulative account of the resources associated with university student mental health.

From an applied perspective, campus physical activity programs may be more relevant to psychological well-being when they create opportunities for mastery, attainable progress, confidence building, constructive feedback, social connection, and adaptive responses to setbacks. Recent reviews of mechanisms and higher-education interventions support incorporating these psychosocial features rather than relying only on exercise volume or intensity [32,33]. The stronger associations observed among students reporting lower perceived social support generate the hypothesis that this subgroup may warrant particular consideration in future intervention research. However, the present cross-sectional findings do not demonstrate differential intervention efficacy. Physical activity programs should complement rather than replace professional assessment and treatment for persistent or severe depressive symptoms.

The study benefited from a multiuniversity sample, adjustment for demographic covariates, and bootstrap estimation of independent, serial, and conditional indirect associations. Nevertheless, several limitations constrain interpretation. The cross-sectional design cannot establish temporal order, and reciprocal associations among depressive symptoms, physical activity, self-esteem, PsyCap, and perceived support remain plausible. All principal variables were measured through self-reports at one time point; although no single factor dominated the measurement variance, Harman’s single-factor test accounted for 30.82% of the variance, and the common method factor model showed only minimal improvement in fit, indicating that method-related covariance cannot be excluded. In addition, the PPQ and PSSS are multidimensional instruments but were represented as single first-order factors to evaluate their global total scores. Given the suboptimal incremental fit of the four-factor measurement model, these unidimensional total-score representations should be regarded as approximate, and findings involving these composite scores should therefore be interpreted cautiously. The sample came from three universities in one Chinese province and included substantially more women and first-year students, limiting broader generalization. Of 670 returned questionnaires, 197 were excluded because one or more required items were missing. Because these questionnaires were incomplete, we could not compare included and excluded respondents on all study variables, which may have introduced selection bias and limited generalizability. In addition, age information, university- and class-level identifiers, and recruitment-mode counts were not retained; consequently, age-related sample characteristics could not be described, and potential clustering within universities or classes could not be formally evaluated, which may have affected the precision of the estimated standard errors. The PARS-3 does not capture activity type, setting, social context, or objectively measured movement, and it was treated as an observed composite rather than a reflective latent construct. We did not conduct a formal unadjusted-versus-adjusted sensitivity analysis. The adjusted model was specified based on theoretical considerations; however, residual confounding from sleep, physical health, socioeconomic circumstances, previous mental health difficulties, and treatment use remains possible.

Future studies should employ longitudinal, intensive repeated measures or experimental designs to test temporal ordering and reciprocal relationships. Combining self-report measures with accelerometry, ecological assessment, clinical symptom measures, and more geographically and demographically diverse samples would also strengthen inference. Intervention studies could then determine whether physical activity programs designed to support self-esteem and PsyCap are especially relevant for students with limited perceived social support.

## 5. Conclusions

Among students recruited from three universities in Guangdong Province, China, physical activity was cross-sectionally associated with fewer depressive symptoms (total association B = −0.175, 95% CI [−0.215, −0.138]), with statistically significant indirect associations involving self-esteem (B = −0.031) and positive psychological capital (B = −0.048), both independently and within the theoretically specified serial model (B = −0.017). The positive psychological capital pathway was larger in absolute magnitude than the serial pathway (difference B = −0.031, 95% CI [−0.048, −0.016]), whereas the self-esteem pathway did not differ significantly from the serial pathway. Perceived social support attenuated these associations, and at high perceived social support, the self-esteem indirect association was no longer statistically significant. These findings highlight the context-dependent relevance of behavioral, intrapersonal, and interpersonal resources within the sampled population. Future longitudinal and experimental studies are needed to determine whether interventions integrating physical activity with psychological resource development improve mental health outcomes and whether their effects differ according to perceived social support.

## Figures and Tables

**Table 1:** Reliability and validity of the psychological scales.

Scale	Factor Loadings	Cronbach α	CR	AVE	VIF
SES	0.617–0.721	0.895	0.897	0.466	1.328
PPQ	0.744–0.818	0.974	0.974	0.592	1.537
SDS	0.584–0.686	0.923	0.927	0.390	-
PSSS	0.706–0.796	0.938	0.939	0.561	1.120
PARS-3 (observed composite)	-	-	-	-	1.272

Abbreviations: PARS-3, Physical Activity Rating Scale-3; SDS, Self-Rating Depression Scale; SES, Rosenberg Self-Esteem Scale; PPQ, Positive Psychological Capital Questionnaire; PSSS, perceived social support; CR, composite reliability; AVE, average variance extracted; VIF, variance inflation factor.

**Table 2:** Fit Indices of the Measurement Models.

Model	χ^2^	df	χ^2^/df	IFI	TLI	CFI	RMSEA	SRMR
Four-factor model	5562.90	2204	2.524	0.845	0.839	0.845	0.057	0.043
Four-factor + method factor (non-orthogonal)	5384.97	2132	2.526	0.851	0.839	0.850	0.057	0.042
Four-factor + method factor (orthogonal)	5583.35	2137	2.613	0.842	0.830	0.841	0.058	-
Three-factor	6971.12	2207	3.159	0.780	0.773	0.780	0.068	0.066
Two-factor a	9980.74	2209	4.518	0.642	0.629	0.641	0.086	0.105
Two-factor b	9920.32	2209	4.491	0.645	0.632	0.643	0.086	0.101
One-factor	12,325.27	2210	5.577	0.534	0.518	0.532	0.098	0.125

Note. *n* = 473; no missing values. All models were estimated with maximum likelihood using semopy 2.3.11 (Python). SRMR values were obtained from lavaan cross-validation because semopy does not report SRMR. The method factor loaded on all 68 items. In the non-orthogonal model, the method-factor covariances with the four trait factors were freely estimated (df = 2132). In the orthogonal sensitivity model, these covariances were fixed to zero and the method-factor variance was fixed to 1 (df = 2137). Both method-factor models showed estimation warnings and are therefore reported as a supplementary check. The two-factor model combined SES, PPQ, and PSSS vs. SDS; model b combined SES and PPQ vs. SDS and PSSS. The three-factor model combined SES and PPQ, with SDS and PSSS separately. The four-factor model was retained as the final measurement model; CFI/TLI/IFI were below 0.90, whereas RMSEA and SRMR were acceptable, indicating relative support for the four-factor structure, but overall absolute fit was mixed.

**Table 3:** Descriptive statistics and Pearson correlations among study variables.

Variable	M	SD	1	2	3	4	5
PA	32.63	21.98	1				
SES	26.22	5.55	0.323	1			
PPQ	104.65	26.14	0.438	0.465	1		
SDS	47.30	10.43	−0.369	−0.398	−0.475	1	
PSSS	46.78	11.72	0.145	0.242	0.304	−0.255	1

Note. PA = physical activity (PARS-3 product score, Winsorized at the 1st and 99th percentiles); SES = self-esteem; PPQ = positive psychological capital; SDS = depressive symptoms; PSSS = perceived social support. All correlations were significant at *p* < 0.01; the PA–PSSS correlation was weaker (r = 0.145, *p* = 0.002). *n* = 473.

**Table 4:** Indirect effects and pairwise comparisons.

Effect	Path	B	Boot SE	95% Percentile CI
Total effect	PA → SDS	−0.175	0.020	[−0.215, −0.138]
Direct effect	PA → SDS (c′)	−0.079	-	[−0.120, −0.039]
Total indirect	Sum of Ind1 + Ind2 + Ind3	−0.097	0.014	[−0.126, −0.070]
Ind1	PA → SES → SDS	−0.031	0.009	[−0.049, −0.016]
Ind2	PA → PPQ → SDS	−0.048	0.009	[−0.068, −0.031]
Ind3	PA → SES → PPQ → SDS	−0.017	0.004	[−0.026, −0.010]
D1 = Ind1 − Ind2		0.017	0.013	[−0.009, 0.043]
D2 = Ind1 − Ind3		−0.014	0.009	[−0.032, 0.003]
D3 = Ind2 − Ind3		−0.031	0.008	[−0.048, −0.016]

Note. Bootstrap *n* = 5000. 95% CIs excluding zero indicate significant effects. Ind1, Ind2, and Ind3 were all significant; D1 and D2 CIs included zero (non-significant), whereas D3 excluded zero, indicating that the PPQ-specific indirect path was significantly stronger than the serial path. Total indirect effect may differ from the sum of specific indirect effects by 0.001 due to rounding. The direct effect was estimated using OLS standard errors; bootstrap SE is not reported.

**Table 5:** Unstandardized coefficients for the serial mediation equations and the simultaneous moderated outcome equation.

Equation/Predictor	B	SE	t	*p*	95% CI
SES equation (M1; R^2^ = 0.117)					
PA_winsor → SES (a_1_)	0.083	0.011	7.55	<0.001	[0.061, 0.105]
Gender (1 = male)	0.301	0.581	0.52	0.605	[−0.841, 1.442]
Grade (1 = second/third-year)	−0.503	0.662	−0.76	0.448	[−1.803, 0.797]
Hometown (1 = urban)	1.231	0.499	2.47	0.014	[0.250, 2.211]
PPQ equation (M2; R^2^ = 0.319)					
PA_winsor → PPQ (a_2_)	0.390	0.048	8.09	<0.001	[0.295, 0.485]
SES → PPQ (d_21_)	1.647	0.191	8.61	<0.001	[1.271, 2.024]
Gender	3.052	2.405	1.27	0.205	[−1.674, 7.779]
Grade	−3.172	2.741	−1.16	0.248	[−8.559, 2.214]
Hometown	3.941	2.079	1.90	0.059	[−0.144, 8.027]
SDS equation (Y; R^2^ = 0.291)					
PA_winsor → SDS (c′)	−0.079	0.021	−3.76	<0.001	[−0.120, −0.038]
SES → SDS (b_1_)	−0.378	0.084	−4.51	<0.001	[−0.543, −0.214]
PPQ → SDS (b_2_)	−0.124	0.019	−6.58	<0.001	[−0.161, −0.087]
Gender	−0.566	0.981	−0.58	0.564	[−2.493, 1.362]
Grade	−1.387	1.117	−1.24	0.215	[−3.583, 0.809]
Hometown	0.321	0.850	0.38	0.706	[−1.349, 1.991]

Note. Continuous predictors were mean-centered before the interaction terms were created. The SES and PPQ equations correspond to the serial mediation model. The SDS equation represents the simultaneous moderated outcome model in which PA × PSSS, SES × PSSS, and PPQ × PSSS were entered concurrently. Gender, grade, and hometown were included as covariates in all equations. For the SDS outcome equation, R^2^ = 0.449, adjusted R^2^ = 0.437, F(10, 462) = 37.589, *p* < 0.001, *n* = 473. PA_winsor = Winsorized physical activity; SES = self-esteem; PPQ = positive psychological capital; PSSS = perceived social support; SDS = depressive symptoms.

**Table 6:** Indices of Moderated Mediation.

Index	Path	Point Estimate	Boot SE	95% Percentile CI
a_1_ × i_2_	PA → SES → SDS	0.0021	0.0005	[0.0011, 0.0032]
a_2_ × i_3_	PA → PPQ → SDS	0.0024	0.0006	[0.0013, 0.0038]
a_1_ × d_21_ × i_3_	PA → SES → PsyCap → SDS	0.0008	0.0003	[0.0004, 0.0014]

Note. All three CIs excluded zero (lower bounds strictly > 0), indicating that social support significantly moderated all three indirect pathways. Coefficients are reported to four decimal places.

**Table 7:** Conditional indirect effects at low, mean, and high PSSS.

Indirect Path	PSSS Level	B	Boot SE	95% Percentile CI
PA → SES → SDS	Low	−0.054	0.011	[−0.075, −0.034]
	Mean	−0.029	0.007	[−0.044, −0.016]
	High	−0.004	0.009	[−0.022, 0.013]
PA → PPQ → SDS	Low	−0.077	0.013	[−0.104, −0.054]
	Mean	−0.048	0.008	[−0.065, −0.034]
	High	−0.020	0.009	[−0.039, −0.003]
PA → SES → PPQ → SDS	Low	−0.027	0.006	[−0.040, −0.017]
	Mean	−0.017	0.004	[−0.026, −0.010]
	High	−0.007	0.003	[−0.014, −0.001]

Note. Low = PSSS mean − 1 SD (M = 46.78, SD = 11.72); mean = centered PSSS = 0; high = mean + 1 SD. Bootstrap *n* = 5000, percentile CIs. At high PSSS, the SES-mediated path (Ind1) was no longer significant (CI included zero), whereas the PPQ-mediated and serial paths remained significant.

**Table 8:** Simple slopes of PA, SES, and PPQ predicting SDS at low, mean, and high PSSS.

Predictor	PSSS Level	B	SE	95% CI
PA_winsor	Low	−0.143	0.028	[−0.198, −0.089]
	Mean	−0.093	0.019	[−0.129, −0.057]
	High	−0.043	0.026	[−0.094, 0.008]
SES	Low	−0.652	0.100	[−0.848, −0.455]
	Mean	−0.352	0.075	[−0.499, −0.205]
	High	−0.052	0.106	[−0.260, 0.155]
PPQ	Low	−0.197	0.024	[−0.243, −0.151]
	Mean	−0.124	0.017	[−0.158, −0.091]
	High	−0.051	0.024	[−0.099, −0.004]

Note. Simple slopes are unstandardized conditional coefficients from the custom moderated outcome equation in PROCESS v4.1, equivalent to Model 89. The 95% CIs for simple slopes are based on model standard errors. Bootstrap *n* = 5000 with percentile-based 95% CI was used for indirect and conditional indirect associations. PSSS levels: Low = mean − 1 SD = 35.063; mean = 46.784; high = mean + 1 SD = 58.506. For PA and SES, the high-PSSS simple slopes were not statistically significant; the PsyCap → SDS slope remained significant at all three levels.

## Data Availability

The data presented in this study are available on request from the corresponding author. The data are not publicly available due to privacy and ethical restrictions.

## Supplementary Materials

The following supporting information can be downloaded at: https://www.mdpi.com/article/10.3390/healthcare14203484/s1, Table S1: Latent factor correlation matrix with AVE on the diagonal; Table S2: Regression diagnostics for the final SDS model; Table S3: Sensitivity analysis: PA untransformed vs. winsorized.

## Abbreviations

The following abbreviations are used in this manuscript:

H	Hypothesis
PA	Physical Activity
PARS-3	Physical Activity Rating Scale-3
PPQ	Positive Psychological Capital Questionnaire
PSSS	Perceived Social Support Scale
PsyCap	Positive Psychological Capital
SDS	Self-Rating Depression Scale
SE	Self-Esteem
SES	Self-Esteem Scale
