# Supplementary material for: Physical Activity and Depressive Symptoms in Guangdong University Students: A Cross-Sectional Moderated Serial Indirect-Association Model Involving Self-Esteem, Positive Psychological Capital, and Perceived Social Support
Source: Healthcare (Basel). 2026 Oct 16;14(20):3484. doi: 10.3390/healthcare14203484 (PMC13704037; doi:10.3390/healthcare14203484)
Supplement: Supplementary file 1 [file healthcare-14-03484-s001.zip › healthcare-4572966-supplementary.pdf]

Supplementary Tables

Table S1. Latent factor correlation matrix with AVE on the diagonal.

Construct	SES	PPQ	SDS	PSSS
SES	0.682			
PPQ	0.494	0.769		
SDS	−0.436	−0.500	0.625	
PSSS	0.267	0.319	−0.271	0.749

Note. Diagonal entries are AVE (square roots of average variance extracted); off-diagonal entries are latent factor correlations from the four-factor CFA. All diagonal values exceed the absolute values of off-diagonal correlations, satisfying Fornell-Larcker discriminant validity.

Table S2. Regression diagnostics for the final SDS model.

Diagnostic	Value	Threshold	Cases exceeding threshold
Max |studentized residual|	3.709	|z| > 3	1 case (index 299)
Max leverage (hat value)	0.153	2(k+1)/n = 0.0465	42 cases
Max Cook's distance	0.056	4/n = 0.0085	24 cases (none > 1)

Note. Twenty-four cases exceeded the 4/n screening cutoff, but the maximum Cook's D was 0.056, and no observation materially altered the focal estimates.

Table S3. Sensitivity analysis: PA untransformed vs. winsorized.

Quantity	PA untransformed	PA winsorized	Relative difference
PA mean	32.81	32.63	−0.53%
PA SD	22.68	21.98	−3.07%
r(PA, SDS)	−0.363	−0.369	Δ = −0.006
Total effect	−0.167	−0.175	+5.1%
Direct effect (c')	−0.074	−0.079	+6.2%
Total indirect	−0.093	−0.097	+4.2%
Ind1	−0.030	−0.031	+5.3%

Quantity	PA untransformed	PA winsorized	Relative difference
Ind2	−0.047	−0.048	+3.3%
Ind3	−0.016	−0.017	+4.8%
PA × PSSS (i_1)	0.0040	0.0043	+7.7%

Note. Eight cases were winsorized (four below the 1st percentile, four above the 99th; cut points = 2.0 and 96.0). N = 473 in both analyses. All effect directions and significance conclusions were unchanged; magnitudes differed by < 8%, and SES/PPQ/PSSS-related coefficients were essentially unaffected (< 1%). Final results reported in the main text are based on the winsorized data.
